# Metastatic Melanoma to the Stomach: A Rare Case of Tumor Burden Beyond the Skin

**DOI:** 10.7759/cureus.87636

**Published:** 2025-07-09

**Authors:** Akhil Adla, Rahul Reddy Tirumalareddy, Peter Snell

**Affiliations:** 1 Department of Medicine, University of Tennessee, Memphis, USA; 2 Department of Internal Medicine, The University of Tennessee Health Science Center, Memphis, USA; 3 Department of Gastroenterology, Gastro One, Memphis, USA

**Keywords:** adult gastroenterology, egd, malignant melanoma metastasis, mortality in melanoma, stomach neoplasm

## Abstract

Metastatic melanoma is a rare occurrence and is a challenge to diagnose based on the location of spread. The increased incidence in the United States (US) can be attributed to the popularity of tanning beds, an aging population, climate change, public awareness, and better diagnostic tools. Despite the large number of melanoma cases, five-year survival rates remain high; however, survival rates dramatically change with metastatic disease. Melanoma commonly spreads to other skin sites, lymph nodes, the liver, bones, and the brain. We present a rare instance of metastatic melanoma in the stomach in an 81-year-old male patient with nonspecific gastrointestinal (GI) symptoms, including five years of dysphagia, weight loss, and coughing spells. This case discusses the prognostic implications of metastatic disease, the importance of proper staining with biopsy samples, treatment, and highlights the importance of recurring disease surveillance.

## Introduction

Melanoma is a cancer caused by the overgrowth of melanocytes. Melanocytes are mostly in the skin and uvea of the eyes. Melanocytes make the pigment that is transferred to keratinocytes, which enables photoprotection from UV radiation [1]. The risk of melanoma increases in a linear fashion as people age until the age of 50, as UV damage accumulates over time [1]. Melanoma incidence has been growing for the last six decades and has increased by 320% since 1975 in both women and men [2]. Despite the large number of new yearly cases, only 4% of melanoma cases become metastatic to distant sites [3]. With the growth in incidence, there is a larger melanoma burden on the healthcare system and higher cases of metastatic spread. The most common sites of spread are the skin, lymph nodes, liver, and the central nervous system. Gastrointestinal (GI) spread is rare, with less than 4% of total metastatic melanoma cases [4]. In instances of GI metastasis, the small bowel, colon, and anorectum accounted for the vast majority of cases, the stomach being exceedingly rare. The prognosis with metastatic melanoma is significantly worse than that of melanoma without spread. The five-year survival for metastatic melanoma is 30%, versus localized, which is over 98.2% [3]. Since spread to the stomach is exceedingly rare, the findings from esophagogastroduodenoscopy (EGD) results often erroneously label it as cancer originating from gastric tissue; however, it is essential to biopsy and conduct proper staining to verify if it is a gastric primary tumor or a secondary spread from melanoma. This case report highlights the clinical presentation and diagnosis approach to overcome the challenges of diagnosing metastatic melanoma to the stomach.

## Case presentation

**Figure 1:**
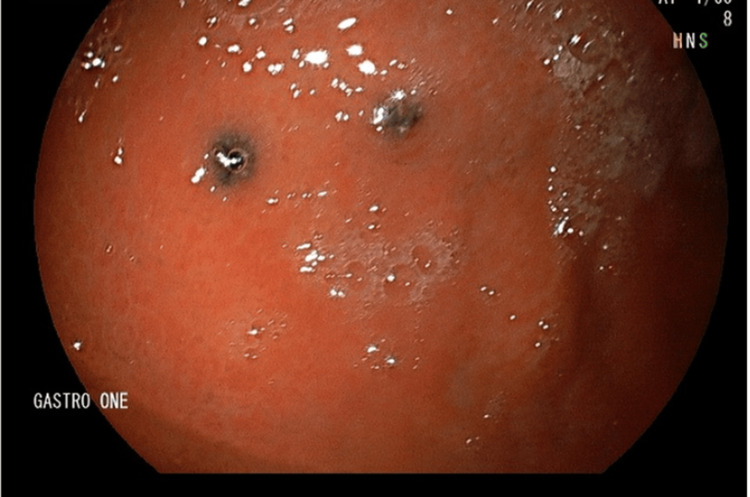
Multiple (2-3 mm in diameter) dark pigmented punctate ulcers in the gastric body.

**Figure 2:**
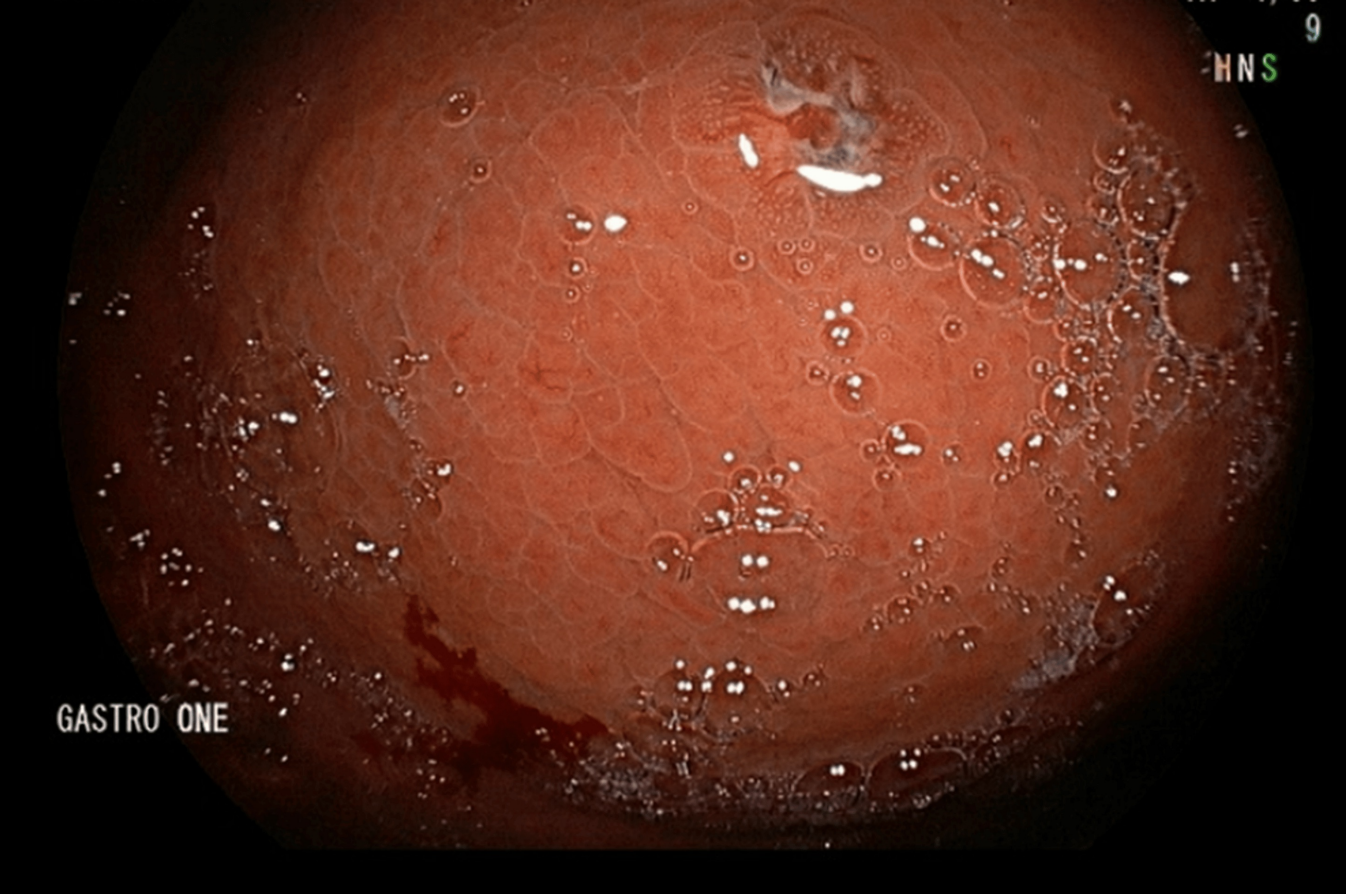
Small 3 cm dark pigmented punctate ulcer in the gastric fundus.

**Figure 3:**
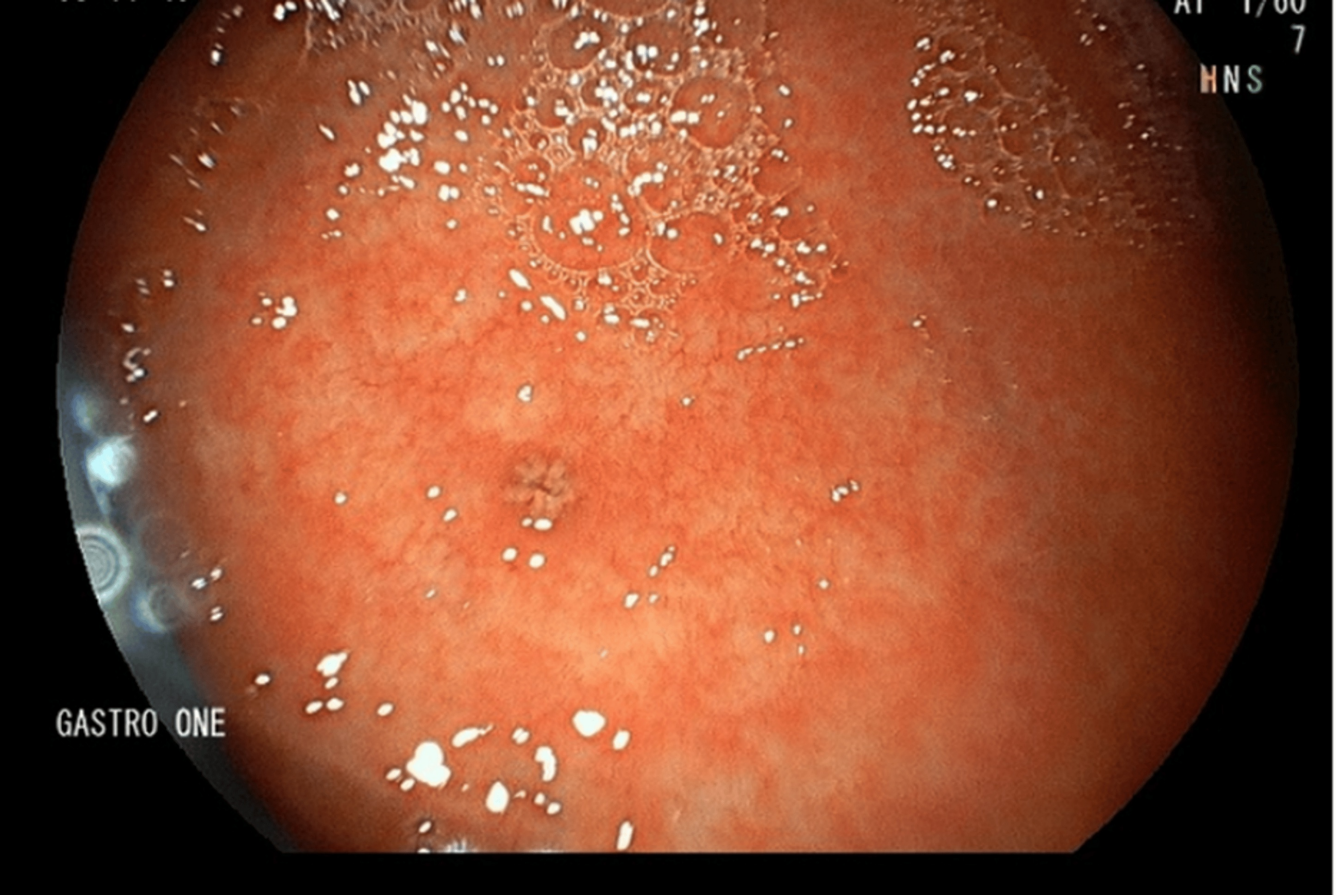
Small 1-2 dark pigmented punctate ulcers in the gastric body.

We present an 81-year-old male with a past medical history of esophagitis, gastroesophageal reflux disease (GERD), remote history of melanomas in the left ear and scalp, Barrett’s esophagus, hypothyroidism, colonic polyps, diabetes, hypertension, and bulging cervical discs who presented to the GI clinic with five years of dysphagia that has been gradually worsening along with debilitating coughing spells that would continue until food made it to the stomach. Of note, the patient 20 years ago had cervical plate insertion for bulging discs and was told the plate was pushing against his esophagus, leading to his coughing spells. The patient had over 40 pounds of weight loss in the last five years due to progressive dysphagia and coughing spells. He was only tolerating one meal a day because of the severity of his symptoms. The patient denied any shortness of breath, chest pain, bloating, nausea, vomiting, abdominal pain, hematochezia, or melena. He was having regular, twice-a-day bowel movements but with liquid consistency for the last several years and had a colonoscopy 10 years ago without any concerning findings. He underwent an EGD 15 years ago with dilation and was given pantoprazole to take daily. On physical exam at the clinic, the patient had clear lung sounds bilaterally, no oropharyngeal findings, no abdominal pain, distension, or rebound tenderness, and normal bowel sounds. The patient, when visiting the GI clinic, had elevated blood pressure and was sent to a primary care visit to better control blood pressure before undergoing an EGD and colonoscopy. A celiac profile, GI stool profile, and high-fiber diet were recommended at the initial visit. The patient was scheduled for EGD and colonoscopy due to his symptoms of weight loss and dysphagia. Stool and celiac without any abnormal findings, but the patient continued to experience the symptoms. After blood pressure was stabilized by primary care, the patient underwent both procedures. Colonoscopy found several adenomas that were removed, and biopsies were positive for mastocytic enterocolitis. EGD discovered several findings, including a soft esophageal extrinsic impression in the middle third of the esophagus at 20 cm from the incisors, a short segment of salmon-colored mucosa concerning for Barrett’s esophagus, and several non-bleeding punctate ulcerations with dark discolorations (Figures 1-3). The biopsies were stained with S-100, Melan-A, PAS/Alcian blue stain, and pan keratin. Pathology showed a poorly differentiated neoplasm that is consistent with melanoma (Table 1). The patient was referred to hematology/oncology for melanoma. A pan-body scan was completed for staging, which revealed widely metastatic disease (Table 1). Hypermetabolic nodule in the left preauricular area with bilateral malignant effusions, right hepatic metastases, splenic metastases, and metastasis to the lateral right thigh. The patient was found to be positive for NRAS mutation and TMB 14.18, and oncology started the patient on nivolumab and relatlimab therapy.

**Table 1:** Summary of the imaging studies and pathology report. CT showing metastatic disease with significant burden in the lungs.

Study	Summary
Pathology Report	Poorly differentiated neoplasm, consistent with melanoma. Histologic sections reveal gastric cardio-fundic mucosa with mild chronic inflammation and poorly differentiated neoplasm with prominent pigment and is reactive for S-100 and Melan A.
CT – PET	Widespread metastatic disease. Hypermetabolic nodule in the left preauricular area. Innumerable bilateral pleural and parenchymal metastases in the lungs along with small bilateral malignant effusions, right hepatic metastases, multiple splenic metastases, and subcentimeter intramuscular metastasis in the lateral right thigh.
MRI Neuro	No evidence of intracranial metastasis

## Discussion

The GI tract is an unusual site for melanoma metastases. Melanoma cells are highly antigenic, allowing them to avoid the immune system, and are highly angiogenic, enabling them to rapidly multiply [5]. Melanoma cells have a greater concentration of oleic acid in their cell membranes, which allows melanoma cells to resist ferroptosis and other means of cell destruction [6]. These distinctive features enable melanoma to spread aggressively, which explains the widespread disease, including metastases to the liver, spleen, GI tract, and lateral thigh in this patient. This case further affirms the importance of recurring surveillance with even a remote history of melanoma to prevent progression. Once metastatic, the prognosis, even with treatments, is significantly reduced. Five-year survival with localized spread is over 98%; regional spread decreases survival to 63%, while distant metastatic disease drops survival to 22.5% [7]. The diagnosis of melanoma can be made with a physical exam and pathology. Imaging modalities like CT can be utilized for metastatic disease, but the gold standard for gastrointestinal (GI) melanoma is endoscopy with biopsies. Positron emission tomography/computed tomography (PET/CT) can provide greater detection and is often utilized as part of staging prior to initiating therapy. GI melanoma can often be misdiagnosed as a GI tumor since GI melanoma can visually appear in ulcerated or polypoid masses with and without pigmentation, making it challenging to diagnose [3]. Staining biopsies with HMB-15, Melan-A, and S-100b is recommended to ensure accurate diagnosis and enable the patient to get the right therapy. S-100b has the highest sensitivity, while HMB-15 and Melan-A are highly specific [8]. Staining or history should be specified to the pathologist, especially when a high index of suspicion exists. History becomes critical for diagnosis since melanoma in the GI tract presents with non-specific symptoms including abdominal pain, dyspepsia, weight loss, dysphagia, hematemesis, and melena. Prior to treatment, the patient must meet diagnostic criteria, including a negative full-body workup, all gastric lesions biopsied, and confirmed melanoma on pathology. Melanoma is treatable in both isolated and metastatic forms. Treatment for skin melanoma is local excision and Mohs micrographic surgery. Treatment of metastatic melanoma varies widely based on the stage of melanoma and the organ system involved. Patients can undergo surgical resections, radiation, and adjuvant therapies for metastatic melanoma. The first line of treatment for unresectable GI melanomas in stage III and beyond is nivolumab and ipilimumab or monotherapy with pembrolizumab [9]. The patient in this case had an extensive spread, which reduced the number of treatment options available. Lactate dehydrogenase (LDH) and S100b are biomarkers utilized currently in prognosis and monitoring melanoma recurrence [1,10]. S100b is a protein released by melanoma cells and has specificity and sensitivity for melanoma [10]. It is plausible to utilize LDH and S100b at the initial visit if there is a high index of suspicion to determine if the workup needs to be expedited, since the presence of both would indicate metastatic disease [11].

## Conclusions

Metastatic melanoma to the GI tract is a rare occurrence that has a dismal prognosis. Early detection remains crucial for avoiding complications and improving the survival rate. This case reaffirms the importance of recurring surveillance in anyone with a history of melanoma, as early recognition can minimize the risk of spread. Diagnosis of metastases requires multimodal testing, including imaging and appropriate pathology staining to verify the diagnosis. Certain metastases, like GI metastasis, can often look like primary solid tumors, and staining is crucial for accurate diagnosis. Blood markers like LDH and S100B can play a role in helping clinicians have a high index of suspicion in any patient with GI symptoms, especially in patients with any previous history of melanoma.
